# Multi-omics analysis revealing the interplay between gut microbiome and the host following opioid use

**DOI:** 10.1080/19490976.2023.2246184

**Published:** 2023-08-23

**Authors:** Udhghatri Kolli, Richa Jalodia, Shamsudheen Moidunny, Praveen Kumar Singh, Yuguang Ban, Junyi Tao, Gonzalo Nathaniel Cantu, Eridania Valdes, Sundaram Ramakrishnan, Sabita Roy

**Affiliations:** a Department of Surgery, University of Miami Miller School of Medicine, Miami, FL, USA; b Department of Public Health Sciences, University of Miami Miller School of Medicine, Miami, Fl, USA

**Keywords:** Opioids, multi-omics, microbial dysbiosis, inflammation, metabolites, barrier dysfunction, host-microbiome interaction, gene expression

## Abstract

Opioid crisis is an ongoing epidemic since the past several decades in the United States. Opioid use-associated microbial dysbiosis is emerging as a key regulator of intestinal homeostasis and behavioral responses to opioid. However, the mechanistic insight into the role of microbial community in modulating host response is unavailable. To uncover the role of opioid-induced dysbiosis in disrupting intestinal homeostasis we utilized whole genome sequencing, untargeted metabolomics, and mRNA sequencing to identify changes in microbiome, metabolome, and host transcriptome respectively. Morphine treatment resulted in significant expansion of *Parasuterella excrementihominis, Burkholderiales bacterium 1_1_47*, *Enterococcus faecalis*, *Enterorhabdus caecimuris* and depletion of *Lactobacillus johnsonii*. These changes correlated with alterations in lipid metabolites and flavonoids. Significant alteration in microbial metabolism (metabolism of lipids, amino acids, vitamins and cofactors) and increased expression of virulence factors and biosynthesis of lipopolysaccharides (LPS) and lipoteichoic acid (LTA) were observed in microbiome of morphine-treated animals. In concurrence with changes in microbiome and metabolome extensive changes in innate and adaptive immune response, lipid metabolism, and gut barrier dysfunction were observed in the host transcriptome. Microbiome depleted mice displayed lower levels of inflammation, immune response and tissue destruction compared to mice harboring a dysbiotic microbiome in response to morphine treatment, thus establishing dysbiotic microbiome as mediator of morphine gut pathophysiology. Integrative analysis of multi-omics data highlighted the associations between *Parasutterella excrementihominis, Burkholderiales bacterium 1_1_47*, *Enterococcus faecalis*, *Enterorhabdus caecimuris* and altered levels of riboflavin, flavonoids, and lipid metabolites including phosphocholines, carnitines, bile acids, and ethanolamines with host gene expression changes involved in inflammation and barrier integrity of intestine. Omic analysis also highlighted the role of probiotic bacteria *Lactobacillus johnsonii*, metabolites flavonoids and riboflavin that were depleted with morphine as important factors for intestinal homeostasis. This study presents for the first time ever an interactive view of morphine-induced changes in microbial metabolism, strain level gut microbiome analysis and comprehensive view of changes in gut transcriptome. We also identified areas of potential therapeutic interventions to limit microbial dysbiosis and present a unique resource to the opioid research community.

## Introduction

Opioid drugs are the gold standard for managing peri- and post-operative pain. However, long-term use of opioids for pain management is limited by adverse side effects, including tolerance, addiction, hyperalgesia, organ damage, cognitive impairments, and adverse effects on respiratory and gastrointestinal systems. Despite these known consequences, a staggering number of opioid prescriptions (i.e., 142,816,781) were dispensed in the year 2020 alone in the United States.^1^ In addition, the total number of opioid-involved overdose deaths rose from 21,088 in 2010 to 68,630 in 2020.^2^ Taken together, there is a critical need for understanding the etiology of opioid use disorder and developing adjunctive therapies to minimize the side effects of opioid drug use.

Several recent studies have shown adverse effects of opioids use on the gastrointestinal system, impacting the intestinal barrier function,^3^ inducing microbial dysbiosis,^4–7^ and modulating opioid induced bowel dysfunction.^8^ Notably, opioid-induced gut microbial dysbiosis is observed in both preclinical and clinical studies,^4,5,9–12^ with a signature profile that show a depletion of bacteria belonging to genera *Lactobacillus*, *Bifidobacterium*, and *Clostridium*, and an expansion of *Enterococcus faecalis*.^5–7,13^ However, these studies are limited by the low resolution of 16s sequencing technique, that leads to the identification of opioid use associated taxonomic alterations at the genus level. Additionally, clinical studies had relatively small opioid patient cohort and were confounded by polydrug use, alcohol use, and different dietary habits in the study population.

In addition to alterations in the gut microbiome, opioid use is known to modulate the gut metabolome, with alterations observed as early as 24 hours following morphine use.^6^ In cohort studies, altered urinary and fecal metabolites were observed in opioid users^14^ and in methadone-maintained patients.^15^ However, in the absence of metagenomic alteration data and comprehensive analysis of metabolite changes from such studies, the role of the gut microbiome in mediating opioid-associated metabolite changes remain largely unknown.

Growing evidence continue to indicate a crucial role for the gut microbiome and metabolome in regulating neuro inflammation,^16^ development of antinociceptive tolerance,^7,11^ opioid-dependent behaviors, alterations to reward and behavioral responses to opioids.^17,18^ and withdrawal symptoms.^16,19^ Additionally, opioid use and gut microbiome are associated with exacerbated progression of HIV,^12,20,21^ colitis,^22^ chronic cirrhosis,^9^ and increased virulence of hospital acquired infections and sepsis.^23–25^ Despite these advances, the role of opioid-associated changes in microbial composition and metabolism in modulating the host immune response, function and metabolism remains uncharacterized. Thus, it is of importance to comprehensively study the morphine-mediated early changes in gut microenvironment and identify potential therapeutic targets to limit microbial dysbiosis and thus delay the development of opioid-associated comorbidities.

In this study, to bridge these gaps in knowledge we conducted a comprehensive analysis of early changes in gut microenvironment by measuring changes in gut microbial communities (shotgun metagenomics), transcriptional changes in gut tissues (RNA seq) and changes in ileal metabolites (untargeted metabolomics) in mice treated with and without morphine for 16 hours. Separate cohorts of mice treated with antibiotics were used to elucidate the role of microbiome in morphine-mediated changes in the small intestine as demonstrated in study design (Supplementary Fig. S1). Different datasets were integrated to delineate the complex relationship between the microbiome, metabolome, host metabolism and immune response in the context of morphine. This study has identified several new mechanisms and potential therapeutic targets that may help future researchers understand the causal relationship between opioid-induced gut dysbiosis, gut barrier disruption, and the development of opioid use disorder.

## Methods

### Experimental animals

All animal experiments were conducted in accordance with the guidelines of Institutional Animal Care and Use Committee (IACUC). Eight-week-old, pathogen free, C57BL/6 male mice (https://www.jax.org/strain/000664) were purchased from Jackson Laboratories (Bar Harbor, ME, USA). Animals were maintained in a 12-h light/dark cycle, with constant temperature (72 ± 1°F) and 50% humidity with food and tap water provided ad libitum. A maximum of five mouse were housed per cage. All the procedures were conducted according to the guidelines set forth by the National Institute of Health Guide for the Care and Use of Laboratory Animals at the University of Miami.

### Animal treatment and sample harvesting

The animals were anesthetized using isoflurane (Pivetal_®_) and a 25 mg slow-release morphine pellet or placebo pellet was implanted subcutaneously. The pellets were obtained from National Institute on Drug Abuse. All efforts were made to minimize suffering during and after surgery. For depletion of the gut microbiota, a pan-antibiotics + antifungal cocktail [vancomycin 32 (mg/kg), bacitracin (80 mg/kg), metronidazole (80 mg/kg), neomycin (320 mg/kg), and pimaricin (0.192 mg/kg)] was prepared every day in drinking water. The cocktail was administered by oral gavage for 7 days as described previously.^7^ Animals were euthanized using CO_2_ chamber followed by cervical dislocation. Luminal content was flushed out from terminal ileum and flash frozen for metabolomic analysis and metagenomics analysis. Ileum tissue were collected, and flash frozen for RNA extraction and preserved in 10% neutral formalin for histological analysis.

### RNA extraction

RNA was extracted from flash frozen terminal ileum from the control and treatment groups, using RNeasy Plus Universal Mini Kit from Qiagen (catalog No. 73404) following manufacturer’s instructions. Briefly, the tissue was homogenized for 90 sec in TRIzol^TM^ (Invitrogen, catalog no. 15596026) using a MagNA Lyser (Roche Diagnostics), followed by non-enzymatic removal of genomic DNA. High quality RNA was extracted using RNeasy spin columns. Total RNA was quantified by using a Nanodrop and the integrity was assessed by using an Agilent 2100 Bioanalyzer. The RNA samples with RNA integrity number (RIN) greater than 8 were used for subsequent RNA -sequencing.

### Histological evaluation and immunofluorescence staining

Excised ileal section was fixed in 10% neutral buffer formalin for 24 hours and embedded in paraffin. 8 µm section was mounted on microscopy glass slide and stained with hematoxylin and eosin (H&E) for histological evaluation. H&E-stained slides were randomized and coded for histological evaluation in a blinded fashion. For immunofluorescence staining with tight junction protein, fixed ileal sections were deparaffinized using xylene and passed through series of graded alcohol for rehydration. Heat antigen retrieval was performed using citrate antigen retrieval buffer (DAKO) and tissue section was blocked with 5% BSA. Ileal tissue sections were stained with anti-Claudin-1 antibody (Invitrogen, catalog no. 51–9000) at a dilution of 1:100 in PBS + 1%BSA overnight at 4°C. Anti-rabbit Alexa 488 conjugated secondary antibody (Invitrogen, catalog no. A-11008) was added at a dilution of 1:800 for 1 hour at room temperature. Ileal tissue section was mounted under coverslip using ProLong Gold antifade mounting media with DAPI (Invitrogen, catalog no. P36935) and visualized using fluorescence microscope (Leica Microsystems, Germany).

### Real-time PCR

Total RNA from terminal intestinal tissue section was extracted using RNeasy Plus Universal Mini Kit from Qiagen (Catalog No. 73404) and quantified using a Nanodrop (Agilent Technologies). cDNA was synthesized from 2 µg of RNA using High-Capacity cDNA Reverse Transcription Kit (Applied Biosystems^TM^ , Catalogue no. 4368814) as per manufacturer’s protocol. Quantitative real-time PCR (qRT- PCR) was performed on LightCycler^Ⓡ^ 480 II (Roche) using SYBR^Ⓡ^ Green I master mix (Roche). Expression levels of measured genes was normalized to 18S rRNA gene by 2-ΔΔCt method. Primers for gene analysis were purchased from Sigma and list of primers used in the study are provided in Table 1.

**Table 1.** Primer used in the study for small intestine gene expression.

Primer Name	Forward primer (5′- 3′)	Reverse primer (5′- 3′)
IL6	CTCATTCTGCTCTGGAGCCC	CAACTGGATGGAAGTCTCTCTTGC
IL17	GGAGAGCTTCATCTGTGTCTCTG	TTGGCCTCAGTGTTTGGACA
TLR2	CGGACTGTTTCCTTCTGACCA	AGATTTGACGCTTTGTCTGAGGT
TLR4	GCTTGAATCCCTGCATAGAGGTAG	CTTCAAGGGGTTGAAGCTCAGA
IL22	TTGAGGTGTCCAACTTCCAGCA	AGCCGGACGTCTGTGTTGTTA
NLRP3	GATGCTGGAATTAGACAACTG	GTACATTTCACCCAACTGTAG
REG3B	AAGGTGCTCATGTCCTCATC	AAGGTGCTCATGTCCTCATC
REG3G	AAGGTGCTCATGTCCTCATC	AAGGTGCTCATGTCCTCATC
CXCL5	ATTGATCGCTAATTTGGAGG	TGTCACTCCCCAATATTTTC
IL1B	GGCAGGCAGTATCACTCATT	AAGGTGCTCATGTCCTCATC
IL18	AAATGGAGACCTGGAATCAG	CCTCTTACTTCACTGTCTTTG
CXCL17	AAGCAGTGTCCCTGTGAT	TTGCGACTTCCTGTGGTG
MMP13	CTTTAGAGGGAGAAAATTCTGG	CATCATCATAACTCCACACACG
MMP16	CTGACAAGATCCCTCCACCTAC	GTGTTGAAGTTCCCATCACAGA
18S	GTAACCCGTTGAACCCCATT	CCATCCAATCGGTAGTAGCG

### Library preparation and sequencing

mRNA was purified from total RNA from using poly T-magnetic beads and strand specific library was constructed by using NEBNext Ultra RNA library prep kit. After quality control, the libraries were sequenced paired end by using Illumina sequencers for a read length of 150 base pairs. The sequencing service was provided by “Novogene”. The raw data was filtered to remove low quality reads and adaptors. Clean reads were mapped to the mouse transcriptome using “STAR” software. The subsequent differential gene expression analysis was performed using DESeq2 R package (log2 (Fold change) > 1, P adj < .05).^26^ Functional enrichment analysis was performed using “ClusterProfiler”^27^ to identify enriched biological process and pathways including Gene ontology (GO) and KEGG^28^ pathways. Differentially expressed genes were further analyzed using ToppGene^29^ and ToppCluster.^30^ Cytoscape^31^ was utilized for visualization of significantly enriched GOs, pathways and disease. Cytoscape.v3.9.0 was used to visualize the network.

### DNA extraction

DNA was extracted from the luminal content of ileum using DNeasyPowerSoil Kit (Catalogue No. 12888–100) from Qiagen according to manufacturer’s instruction. The extracted DNA was quantified by Qubit 4.0 fluorometer.

### Metagenomic sequencing

The libraries were constructed using Nextra XT DNA library preparation kits. After QC analysis, the libraries were sequenced as 2 × 150bp reads at a depth of 20 million reads on an Illumina NovaSeq6000 sequencer.

### Data analysis and taxonomic profiling

The sequenced reads were uploaded and analyzed using the Cosmos ID cloud platform and bioinformatics pipeline.^32,33^ Briefly, the unassembled reads were analyzed by using data mining K-mer algorithm. Taxonomic assignment was carried out by matching the specific K-mers identified in the sequenced samples to the unique, shared K-mers and marker genes of the reference genomes present in highly curated GenBook^TM^ reference database. A threshold based on internal Cosmos ID statistics was assigned to avoid false positives. Use of this unique K-mer based approach was proven to be accurate in taxonomic assignment up to species, sub species and strain level at multi kingdoms, including bacteria, virus, protists and fungi.

### Relative abundance and diversity matrices

Abundance of each organism was calculated based on the K-mers unique to the organism, their observed frequency and size of the reference genome. The changes in relative abundance at all taxonomic levels was calculated and presented as a percentage. Differently altered taxa were identified by linear discriminant analysis of effect size (LEfSe) using Kruskal-Wallis test with a *p* value < .05 and linear discriminant analysis (LDA) score > 2.0 and represented in a cladogram.^34^ Changes in alpha and beta diversity resulting from the treatment were presented using Shannon and Bray-Curtis dissimilarity matrices. Non-parametric PERMANOVA analysis “adonis” function was utilized for statistical analysis of Bray-Curtis dissimilarity matrices.

### Functional profiling

Metagenomic shotgun reads of control and morphine treatment groups were analyzed using HUMAnN2 2.8.1. to identify bacterial community functional potential.^35^ Briefly, mouse reads were removed by mapping to the mouse genome using Bowtie2. UniRef 90^36^ and MetaCyc^37^ databases were used to annotate the gene family, and pathway abundances respectively. MetaPhlAn2 2.9^38^ was used to map the gene and pathway abundances to bacteria (species, genus or unclassified) in control and morphine treatment groups. Genes were further regrouped in KEGG orthology (KO) and GO using the “humann2_regroup_table” function. Gene and pathway abundances were normalized using read per kilo base to account for gene length and further normalized to total microbial reads. The final values of gene and pathway abundances were presented as number of copies per million (CPM) in log2 scale. Moderated t test calculated using the “limma” function in R, adjusted for multiple hypothesis testing (FDR) was used to compare gene, KO and pathway abundance between the control and morphine treatment groups. The KOs were grouped into higher functional groups using Microbiome Analyst.^39^

### Untargeted metabolomics

Untargeted metabolomic analysis of harvested samples was done by Michigan Regional Comprehensive Metabolomics Resource Core (MRC^2^) (Ann Arbor, MI, USA). Metabolite extraction was done in Methanol: Acetonitrile: Acetone (1:1:1) solution with internal standards.

Samples were reconstituted in solvent containing Methanol and H_2_O (2:98). 100 µl sample was added to 400 µl extraction solvent and reconstituted in 100 µl reconstitution solvent. For untargeted metabolomics, samples were analyzed on 1290 Liquid chromatography, coupled with 6530 qTOF mass spectroscopy (Agilent Technologies, Santa Clara, CA, USA). Same chromatography was used for positive and negative modes. Raw data was processed using MassHunter Qual and ProFinder software (Agilent), and analyzed with MassProfiler Pro package using recursive analysis workflow (Agilent). Data normalization was performed using custom R-scripts (internal to Michigan Regional Comprehensive Metabolomics Resource Core (MRC^2^) (Ann Arbor, MI, USA). 243 annotated compounds were identified in our sample by comparing the observed mass and retention time to the in-house library. Remaining unannotated compounds were listed with their observed mass and retention times.

### Multi-omics integration-network analysis

Integration of multi-omics data from transcriptome, metagenome and metabolomics was done through correlation analysis. Bacteria, significantly altered metabolites (102) and a total of 1075 DEGs (910 upregulated and 165 down regulated genes) involved in inflammatory signaling, tissue damage, lipid signaling were used for integration analysis and are listed in supplementary file 5. Corresponding data frame for calculation of correlations was prepared and processed in R and function *rcorr* was used to compute spearman correlation coefficient for all possible pairs of matrices. The significance of pair wise correlation was determined after adjusting for false discovery rate using Benjamini-Hochberg procedure using Hmisc package in R.^40,41^ Using Spearman correlation, we identified significantly altered metabolites and DEGs whose expression covaried with dysbiotic microbiome. Also included in the network were various DEGs that covaried with changes in metabolites since microbiome can mediate changes in gene expression through production and transformation of various metabolites. All significant correlations greater than or equal to± 0.6 were used to create a core network. Separate sub networks were constructed by selecting for significantly altered bacterial taxa at species level, metabolites and DEGs. Statistically significant positive and negative correlations between taxa-metabolites; taxa-genes that are greater than 0.7 are included in the network, and top 25% of correlations between metabolites and genes were represented in the sub network. Significant correlations were imported to Cytoscape 3.8.2 for visualization of the correlation network.

### Statistical analysis

For microbiome analysis, differently altered taxa were identified by LEfSe^34^ using Kruskal-Wallis test with a *p* value < 0.05 and LDA score > 2.0. Nonparametric PERMANOVA analysis using “adonis” function was utilized for statistical analysis of Bray-Curtis dissimilarity matrices. Changes in relative abundance of microbial strains were computed using non-parametric Mann-Whitney test (*p* value < 0.05) in GraphPad prism. For analysis of functional changes in metagenome, moderated t-test calculated using the “limma” function in R,^42^ adjusted for multiple hypothesis testing (FDR) using Benjamini-Hochberg procedure was used to compare gene and pathway abundance between the control and morphine treatment groups (P adj < .05). The changes in KEGG metabolic pathways between the groups was calculated by T-test after correction for false discovery with a cut off of P adj < 0.05. Changes in expression of virulence factors were computed using Mann-Whitney test (*p* value < 0.05). For metabolite analysis, after inter quantile normalization the changes in metabolite analysis were calculated using moderated T-test with Padj < 0.05. For RNA-sequencing, differential gene expression analysis was performed using DESeq2 R package and genes with an expression of log2 (Fold change) > 1, and P adj < 0.05 (Benjamini-Hochberg correction) were considered significant. For real-time qPCR analysis, significance of altered expression is calculated using non-parametric Mann-Whitney test (*P* value < 0.05) using GraphPad prism. For multi-omics integration network, the significance of correlation between all bacterial species, metabolites and DEGs was calculated using “Hmisc” package^41^ accounting for multiple testing using Benjamini-Hochberg procedure with a P adj < 0.05.

## Results

### Morphine treatment induces taxonomic and functional dysbiosis in small intestinal microbiome

**Figure 1.**
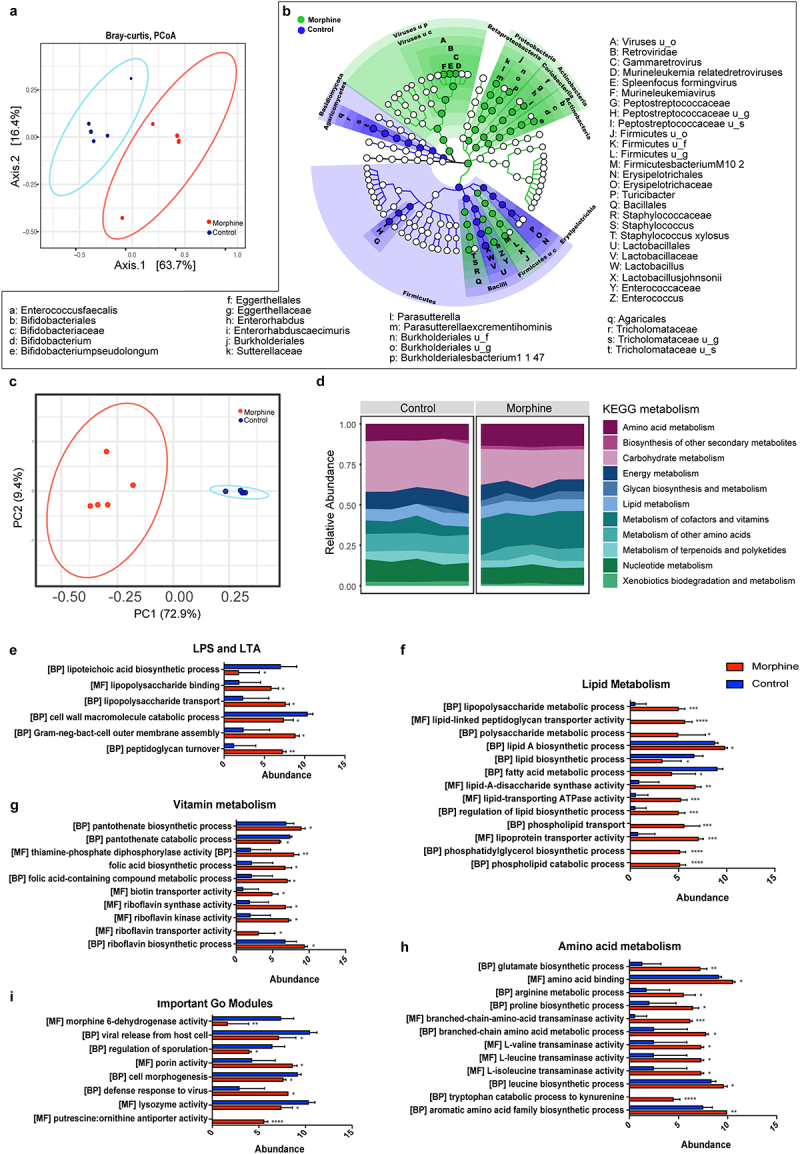
Morphine treatment induces taxonomic and functional dysbiosis in small intestinal microbiome. (a) Beta diversity of phylogenic relative abundance of ileal microbiome at species level in control and morphine group calculated as Bray-Curtis dissimilarity matrix. PERMANOVA analysis (*p* = .026) show significant changes in microbial composition. (b) Cladogram showing the differentially abundant microbial taxa, including bacteria, archaea, virus, fungi identified by LEfSe analysis (*P* < .05 Krushkal-wallis test, LDA > 2.0). Green represents taxa abundant in morphine group compared to control; blue, represents taxa abundant in control group compared to morphine group. (u_s: unclassified species, u_g: unclassified genus, u_f: unclassified family, u_c: unclassified class, u_o: unclassified order) (c) Principal component analysis based on relative abundance of KOs discriminates the functional signature of control and morphine microbiome. (d) Overview of microbiome functional profile based on KEGG metabolism in control and morphine groups. Bar graph showing enrichment of GO modules involved in (e) LPA and LTA biosynthesis, (f) lipid metabolism, (g) vitamin metabolism, (h) amino acid metabolism, and (i) other biologically important GO modules. *n* = 5 mice per group. Data (e, f, g, h, and i) were analyzed using moderated t-test (mean± standard error of mean (SEM), *p adj ≤ .05, **p adj ≤ .01, ***p adj ≤ .001, ****p adj ≤ .0001. *n* = 5 per group.

To understand the effects of morphine treatment on the gut microbiome, we performed whole genome shotgun sequencing of mouse ileal microbiome. We identified a total of 63 taxa at the species level (47 bacteria, 3 fungi, 1 archaeon, 11 viruses, and 2 phages) (Supplementary file1). Beta diversity analysis (Figure 1a) as calculated by Bray-Curtis dissimilarity matrices of the combined taxa shows a distinct clustering of the control and treatment groups. Significantly altered taxa identified by LEfSe with a *p* value < 0.05 and LDA score > 2.0 were represented in cladogram (Figure 1b). Further analysis of the bacterial taxa alone revealed alterations in alpha diversity (Shannon and Simpson indices) and beta diversity (Bray-Curtis) in morphine-treated mice compared to the control mice (Supplementary Fig. S2A, 2B). Morphine treatment resulted in significant expansion of *Parasutterella excrementihominis, Burkholderiales bacterium 1_1_47*, *Enterococcus faecalis*, *Staphylococus xylosus*, *Firmicutes bacterium M10–2*, *Bifidobacterium pseudolongum* and *Enterorhabdus caecimuris* and depletion of *Lactobacillus johnsonii* (Figure 1b). Consistent changes were observed at strain level (Supplementary Fig. S2C).

Furthermore, morphine treatment significantly increased abundance of virus belonging to the genus Gamma retrovirus (Figure 1b). Of note, the alpha and beta diversities for viruses did not change significantly suggesting that morphine caused subtle changes in viral taxa (Supplementary Fig. S2E-F). Among archaea, we observed a significant depletion *of Methanococcus maripaludis* following morphine treatment (Supplementary Fig. S2C).

We next analyzed metagenomic data to identify functional changes by mapping microbial reads using HUMAnN pipeline. The identified reads were then assigned to microbial pathways, GO, and KO. Morphine treatment causes significant alteration to 201 KOs (Supplementary Fig.3; Supplementary file 1). The Principal Coordinate Analysis (PCoA) plot based on the relative abundance of the KOs in control and morphine-treated groups suggest that morphine significantly altered the functional profile of bacterial community (Figure 1c). In addition, grouping of KOs into KEGG metabolic pathways suggest that morphine treatment increased amino acid transport and metabolism, metabolism of cofactors and vitamins, lipid metabolism; and decreased xenobiotics biodegradation and metabolism, and carbohydrate metabolism (Figure 1d; Supplementary file 1). Additionally, we identified 3198 Uniref genes that were grouped into GOs, and we observed significant changes in 460 GO modules (Supplementary file 1). Interestingly, higher abundance of GO modules detected in the metagenome of morphine-treated mice was involved in production of bacterial cell membrane components and transport including lipoteichoic acid (LTA) biosynthetic process, peptidoglycan turnover, gram negative bacteria cell outer membrane assembly, lipopolysaccharide (LPS) binding and transport (Figure 1e). Also, in morphine treatment groups increased expression of genes involved in production of virulence factors in *Bacteroides fragilis* (Supplementary Fig. S2D) were observed, indicating an increased pathogenicity in morphine microbiome.

We also observed an enrichment of GOs involved in lipid metabolism, including lipid A and phosphatidylglycerol biosynthetic process, phospholipid transport and lipoprotein transporter activity and a downregulation of GOs involved in fatty acid metabolic process and lipid biosynthetic process in morphine-treated group compared to the control (Figure 1f), suggesting a dysregulation of lipid metabolism in microbiome of morphine treatment group. Moreover, significant enrichment in GO modules involved in biosynthesis of vitamins (Vitamins B1, B2, B5 and folic acid) (Figure 1g), and biosynthesis of amino acids including glutamate and catabolism of tryptophan to kynurenine (Figure 1h), were observed in morphine microbiome compared to the controls. Furthermore, an increased abundance of GOs involved in defense response to virus was observed in microbiome from morphine-treated mice. Interestingly, we also observed decrease in GOs involved in morphine 6-dehydrogenase activity, viral release from host cell and lysozyme activity in microbiome of morphine-treated mice (Figure 1i). These findings collectively indicate that morphine use results in multi-kingdom level compositional alterations and also induces bacterial functional dysbiosis.

### Morphine use results in broad changes in small intestinal metabolome and highlights changes in lipid metabolism

**Figure 2.**
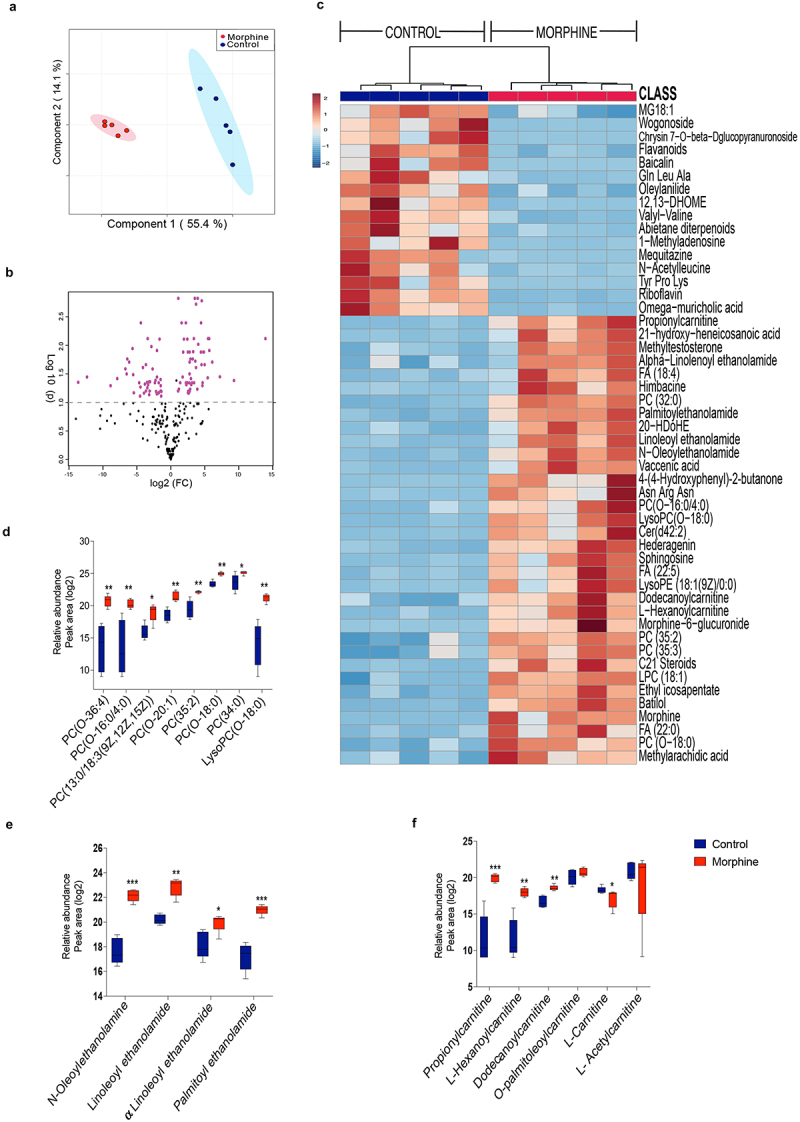
Morphine use results in broad changes in small intestinal metabolome. (a) Score plot showing Partial Least Squares-discriminant analysis (PLS-DA) of morphine and control ileal metabolite profile (variance is explained by PC1 (55.4%) and PC2 (14.1%)). (b) Volcano plot showing most significant metabolites identified by univariate analysis. 115 out of 243 metabolites were significantly different in morphine compared to control samples. (c) Heat map showing top 50 metabolites significantly altered across control and morphine groups. Box plots showing log2 fold changes in relative abundance (Peak area) of (d) phosphocholines, (e) N-acylethanolamines, and (f) carnitines. *n* = 5 per group. Data (d, e, and f) were analyzed using moderated t-test; the box-and-whisker plot indicates the minimum and maximum distribution (whiskers) and the upper and lower quartile limits (box), with the median value shown as a line. *p adj ≤ .05, **p adj ≤ .01, ***p adj ≤ .001, ****p adj ≤ .0001.

To fully characterize the impact of compositional and the functional dysbiosis, we performed metabolite profiling of ileal luminal content of morphine treated and control mice using untargeted Liquid chromatography-mass spectrometry (LC-MS). We have identified and annotated 243 metabolites of which 115 metabolites (53 depleted, 62 enriched) were significantly altered with morphine treatment (Supplementary file 2). The partial least squares-discriminant analysis (PLS_DA) plot differentiated morphine metabolome from the control group (Figure 2a). Metabolite changes with morphine treatment are represented in the volcano plot (Figure 2b, Supplementary file 2). The top 50 altered metabolites are represented as a heat map (Figure 2c). Enrichment of various lipid metabolites including ethanolamines (N-Oleolethanolamine (OEA), Linoleoyl ethanolamide (LEA), Alpha-linolenoyl ethanolamide (αLEA) and Palmitoyl ethanolamide (PEA)), phosphocholines (PCs) (PC(O-20:1), PC (13:0/18:3 (9z,12z,15z), PC(35:2), PC(0–18:0), PC (35:3), Fatty acids (FA) (myristoleic acid, methyl arachidic acid, FA(22:0), FA(20:2), and FA(22:4)), propionylcarnitine, L-hexanoylcarnitine, dodecanoylcarnitine and metabolites of linolic acid, mainly, 12,13-DHOME were observed in morphine metabolome (Figure 2(c-f)). While, depletion of vitamin Riboflavin, flavonoids (genistin, daidzin, naringenin1-O-glucuronide, wogonoside, baicalin), L-carnitine, spermidines and bile acids (BAs) (ursodeoxycholic acid, alpha-muricholic acid, omega-muricholic acid, cholic acid, hyocholic acid, and glycocholic acid), was observed in morphine group (Figure 2(c-f)), and Supplementary file 2). Also, depletion of endocannabinoid receptor ligand 4-Quinolone-3-Carboxamide CB2 Ligand, all-trans-retinoic acid (*at*RA), and zearalenone 4-sulfate were observed in luminal metabolome of morphine group (Supplementary file S2). Furthermore, we observed significant enrichment of morphine metabolite morphine 6-glucoronide (M6G) indicating altered metabolism of morphine (Figure 2c).

### Morphine induced changes in small intestine transcriptome include heightened expression of inflammation associated genes and alterations in lipid metabolism

**Figure 3.**
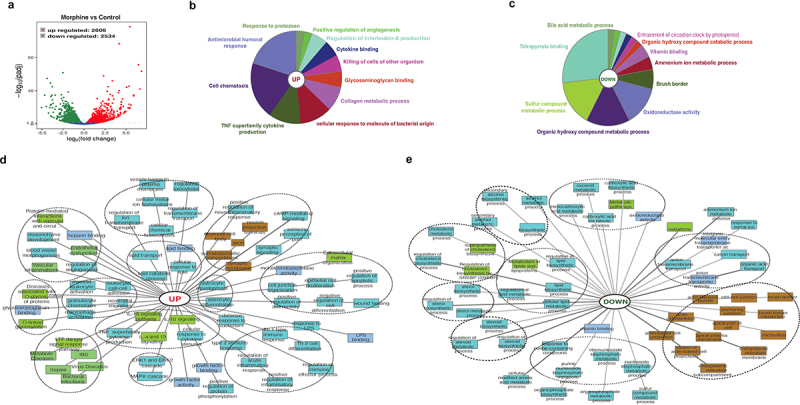
Global overview of small intestine transcriptome changes in morphine treated mice. (a) Volcano plot of the 5140 differentially expressed genes between morphine and control group (Log2 (fold change) > 1; Padj <.005). Functional annotation enrichment analyses of (b) 2606 upregulated and (c) 2534 downregulated genes after morphine using ClueGO charts. Detailed functional annotation enrichment analyses of the (d) 2606 upregulated and (e) 2534 downregulated core genes using ToppGene, ToppCluster, and Cytoscape are shown. GO: biological process (teal), cellular component (brown), and molecular function (blue gray), disease (green). The full list of gene set enrichment results and *P* values are in Supplementary file 3.

We defined core ileal morphine gene expression signature composed of 5140 (2606 upregulated, 2534 downregulated) differentially expressed genes (DEGs) (Figure 3a and Supplementary file 3). ClueGo and ToppCluster were used for gene enrichment analysis. Broadly, ClueGo pie chart for upregulated DEGs showed significant enrichment for immune cell chemotaxis and related humoral and cytokine response (Figure 3b). Simultaneously, enrichment in response against protozoan and bacterial molecules is also observed (Figure 3b). A robust decrease in overall metabolic processes was observed (Figure 3c). ToppCluster analysis was performed on upregulated (Figure 3d) and downregulated genes (Figure 3e) for detailed representation of enriched biological process including significant pathways, GOs and disease. Compared to control, morphine upregulated gene signature shows enrichment of GOs and pathways associated with defense responses such as interleukin signaling pathways, innate and adaptive immune cell activation, response to bacteria and LPS etc. Notably, other upregulated GOs such as MAPK and ERK1/2 signaling cascade, vascular inflammation, endothelial dysfunction, and O-linked glycosylation, were also emphasized in morphine group. The downregulated gene signature for morphine treatment showed a robust decrease in cell-cell junction, actin cytoskeleton and anchoring junction, which were further reflected in the severely damaged tissue morphology observed in H&E stained intestinal section (Supplementary Fig.4A) and tight junction staining (Supplementary Fig.4B) in intestinal sections in morphine treated mice. Using a separate cohort of mice, we validated morphine mediated upregulation of inflammatory markers using real time PCR (Supplementary Fig. 4C). Genes involved in diverse metabolic process, including steroid, lipid, alcohol biosynthesis were downregulated (Figure 3e). Interestingly, upregulation of GOs involved in lipid binding, lipid transport, and lipid catabolic processes was also noted, indicating widespread dysregulation of metabolic process after morphine treatment.

### Morphine mediated changes in murine intestinal transcriptome profiles are influenced by gut microbiota

**Figure 4.**
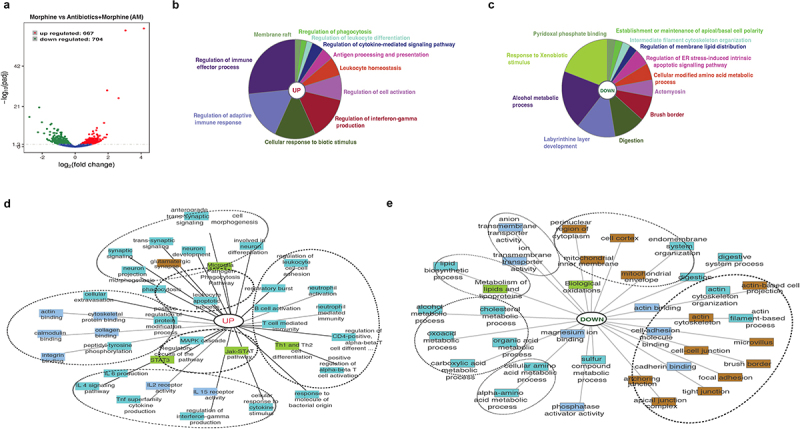
Morphine mediated changes in murine intestinal transcriptome profiles are influenced by gut microbiota. (a) Volcano plot of the 1371 differentially expressed genes between morphine and AM group (Log2 (fold change) > 1; Padj <.005). Functional annotation enrichment analyses of the (b) 667 upregulated and (c) 704 downregulated genes after morphine using ClueGO charts. Detailed functional annotation enrichment analyses of the (d) 667 upregulated and (e) 704 downregulated core genes using ToppGene, ToppCluster, and Cytoscape are shown. GO: biological process (teal), cellular component (brown), and molecular function (blue gray), disease (green). The full list of gene set enrichment results and *P* values are in Supplementary file 4.

To obtain insight into genome wide impact of dysbiotic microbiome characteristic of morphine treatment on the host transcriptome, we compared mRNA expression levels in morphine-treated group and Abx+morphine (AM) group that is treated with morphine in the absence of microbiome. We observed 1371 (667 upregulated and 704 downregulated) DEGs in morphine-treated group compared to AM group (Figure 4a and Supplementary file 4), indicating that the expression of the 1371 genes was mediated by morphine microbiome. Functional annotation enrichment analysis was performed on all significant DEGs (*p* ≤ 0.05) using ClueGo, ToppGene and ToppCluster as mentioned previously. ClueGo pie chart depicts enrichment of various immune regulatory processes as well as cellular response to biotic stimuli such as molecules of bacterial, viral or fungal origin in morphine group (Figure 4b). A simultaneous decrease in response to xenobiotic stimulus was also observed after morphine treatment (Figure 4c). Further analysis on DEGs using ToppCluster analysis revealed an increase in innate and adaptive immune response as well as cytokine signaling in morphine group compared to AM group implicating dysbiotic microbiome as a mediator of inflammation (Figure 4d). The role of gut microbiota in driving inflammation following morphine treatment was further validated using germ free (GF) mouse, where morphine mediated upregulation of pattern recognition receptor (*Tlr4*), cytokines (*Il6*, *Il1β*, *Il18*, *Tnfα*), chemokines (*Cxcl1*, *Cxcl2*, *Cxcl17*) and matrix metallopeptidase 16 (*Mmp16*) involved in tissue damage was not observed (Supplementary Fig. S5) in GF mice.

Several metabolic process pathways including amino-acid metabolic processes, alcohol metabolic processes, lipids and lipoprotein metabolic processes were downregulated robustly (Figure 4e) in morphine group compared to AM group, indicating the role of microbiome in these metabolic processes. Another significant downregulation was observed in cellular components involved with intestinal permeability such as tight junction, cell-cell junction, anchoring junction, actin cytoskeleton (Figure 4e). Taken together, the comparative analysis of gene expression patterns across the morphine group and AM group identified dysbiotic microbiome as a mediator for inflammation and metabolic dysregulation.

### Systematic molecular correlations across multi-omes integrating morphine mediated changes in microbiome, metabolome, and host transcriptome

**Figure 5.**
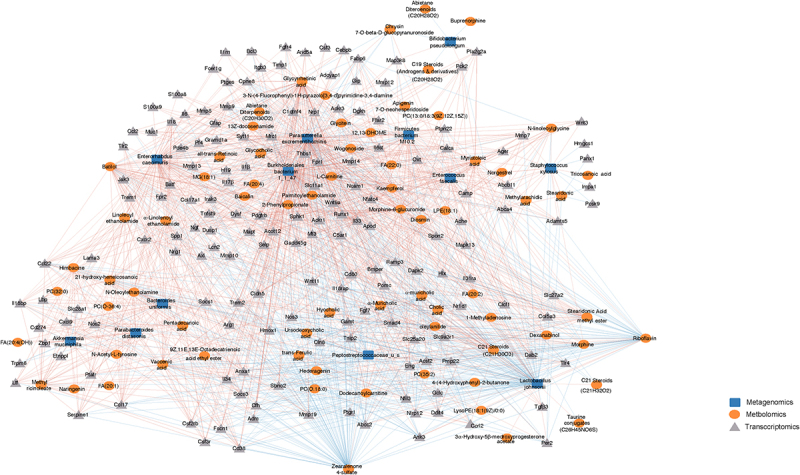
Systematic molecular correlations across multi-omes integrating morphine mediated changes in microbiome, metabolome, and host transcriptome. Host microbiome interaction network (Spearman coefficient ≥ ± 0.7) showing significant correlations between microbiome, metabolites and DEGs (P adj ≤ 0.05). Microbial species, metabolites, and DEGs are represented as nodes; microbiome (blue square), metabolite (orange circle), DEGs (gray triangle). Lines represent statistically significant correlations and are colored red for positive and blue for negative correlations.

Next, to identify the direct microbial-host interactions and indirect interactions mediated by metabolites, we constructed a large-scale interaction network spanning the three omics measurements. Using Spearman correlation, we identified metabolites and DEGs whose expression covaried with changes in microbiome. Also included were various DEGs that covaried with changes in metabolites. In total, we identified 15,617 significant correlations between the three data sets (Supplementary file 5). Filtered network was generated for visualization by integrating the significant Spearman correlations between taxa, metabolites, and DEGs (Figure 5). The resulting network contained 1672 significant correlations, including both positive and negative correlations value greater than 0.7 (Padj <0.05) between 251 nodes from three measurement types (Supplementary file 6).

The bacteria *P. excrementihominis* and *B. bacterium 1_1_47* were well connected in the network, accounted for large number of the interactions, and were associated with 76% and 75% of nodes respectively. *P. excrementihominis* and *B. bacterium 1_1_47* positively correlated with M6G, ethanolamines, fatty acids, phosphocholines and had large number of positive correlations with many genes involved in inflammatory and immune responses (Figure 5, Supplementary Fig. S6A). Pathogenic bacterial taxa *E. faecalis* and *S. xylosus* positively correlated with long chain fatty acid (LCFA) metabolites such as myristoleic acid, tricosanoic acid, methyl arachidic acid, eicosadienoic acid, and docosanoic acid. *E. faecalis* also correlated with changes in ethanolamine. *E. faecalis* and *S. xylosus*, and LCFAs correlated with genes involved in lipid binding, transport, localization, and catabolic processes, implicating these taxa in dysfunction of lipid metabolism. Increased N-acylethanolamines (NAEs) levels were negatively corelated with *L. johnsonii* and *Peptostreptococcaceae* and had strong positive correlations to *P. excrementihominis*, *B. bacterium 1_1_47*, *E. faecalis, E. caecimuris*, and *A. municiphila*. Interestingly NAEs also had strong positive correlations to DEGs involved in inflammatory response including *Il6, Tlr4, Tlr2, Nlrp3, Nos2, Ccrl2, Cxcl9, Cxcr2 Ccl17, Ccl22*, etc. (Supplementary Fig. S6B).

The probiotic bacteria *L. johnsonii* and commensal *Peptstreptococcaceae* of unclassified species had major number of negative correlations accounting to 44% and 9% of negative interactions respectively. We also observed strong negative correlations between probiotic bacteria *L. johnsonii* and several intestinal metabolites including phosphocholines (PC (32:0), PC (0–18:0), PC (35:2)), flavonoids (batilol and hederagenin), ethanolamines and FA (20:4) (Figure 5, Supplementary Fig. 5A). Moreover, positive correlations were observed between *L. johnsonii* and several 1° and 2° BAs (Supplementary file 5) as well as with riboflavin. *L. johnsonii* positively correlated with genes involved in metabolism of lipids and lipid biosynthesis pathways including fatty acid-binding proteins (*Fabp6*) 6, acyl-CoA synthetase family member 2 (*Acsf2*), and per2-circadian rhythm (*Per2*) as well as genes involved in cellular junction such as *Abcc2*, *Ank3*, *Pmp22*, *Mmp7, Mmp9, Mmp10, Mmp19* etc. (Figure 5). Also, *L. johnsonii* and *Peptostreptococcaceae* negatively correlated with the upregulated genes involved immune cell chemotaxis and cellular response to cytokine stimulus.

The metabolites riboflavin, zearoline 4-sulfate, M6G, flavanoids, lipid metabolites including fatty acids, phosphocholines, ethanolamines, along with bile acids and carnitines were featured prominently in the network and showed strong correlations to changes in bacterial species and host transcription highlighting metabolites as important mediators of host microbiome interactions.

## Discussion

The premise of our work was to 1) comprehensively study the morphine treatment associated changes in ileal microbiome composition and metabolic function. 2) Define the consequence of microbial dysfunction on the host by utilizing transcriptomics and metabolomics. 3) Leverage our findings to identify interventional therapeutic strategies to limit microbial dysbiosis implicated in the development of inflammation.

This is the first study to demonstrate morphine associated multi-kingdom level dysbiosis that was characterized by taxonomic alterations in bacteria, archea, virus, and fungi. Significant enrichment of *E. faecalis*, *S. xylosus*, *E. caecimuris*, *P. excrementihominis* and *Burkholderiales bacterium* were observed in the morphine microbiome. Similar microbial perturbations were previously observed in various disease conditions. For example, *E. caecimuris*, was first characterized from a mouse model of spontaneous colitis^43^ and is associated with dysbiosis in Crohn’s disease (CD) and autism spectrum disorder.^44,45^ *Parasutterella spp*. was previously shown to be abundant in the ileal submucosa of CD patients,^46,47^ in dysbiotic microbiome of hypertriglyceridemia-related acute pancreatitis,^48^ and in stool samples of inflammatory bowel syndrome patients,^49^ and increased abundance of *Parasutterella spp*. positively correlated to intestinal inflammation.^49^ Consistent with these findings, the enriched bacteria that include *P. excrementihominis, B. bacterium*, and *E. caecimuris* strongly correlated with upregulated genes involved in immune cell chemotaxis and cellular response to cytokine stimulus highlighting the role of these enriched pathobionts in inflammation. Moreover, several of these genes involved in the cellular response to cytokine, including *Il18*, *Il33*, *Il18bp*, *Il7r*, *Il17rc*, *Il4ra*, *Il2rg*, *Il10ra*, *Il2rb*, *Il15ra*, *Il2ra*, and *Nfil3* showed significant downregulation in AM group compared to the morphine group. These findings clearly indicate that morphine-induced microbial dysbiosis leads to the initiation of host immune response and intestinal inflammation.

Significant depletion of *L. johnsonii NCC533* observed in morphine-treated samples corroborates with previous studies showing depletion of bacterial genus *Lactobacillus* in rodent models and human opioid studies.^10,11,18^ Decrease in commensal bacteria might be facilitating the expansion of pathogenic bacteria as observed in morphine microbiome. In support of this, *L. johnsonii* was also shown to inhibit the growth of pathogens like *E. faecalis*, *E. coli*, and *Salmonella enterica* in coculture.^50^ *Lactobacillus* can also provide protection against pathogenic invasion by enhancing and maintaining intestinal barrier integrity through regulation of tight junction proteins.^51–54^ Our integration data is in accordance with these findings and show a positive correlation of *L. johnsonii* with intestinal barrier related cellular components such as cell-cell junction, brush border membrane, tight junction, and anchoring junction proteins. While it is negatively correlated with genes involved in inflammatory response. Interestingly, the treatment with probiotic cocktail consisting of *Lactobacillus* has significantly reduced morphine-induced gut epithelial barrier disruption, decreased intestinal inflammation, and development of analgesic tolerance,^7^ thus highlighting the protective role of *Lactobacillus*.

Metagenomic functional analysis provided much-needed insight into the changes in the functional potential of the gut bacteria after morphine treatment. Notably, in morphine treatment groups, bacteria displayed an increased potential for LPS, LTA biosynthesis, and increased expression of virulence factors. These findings collectively indicate a taxonomic shift toward a proinflammatory and pathogenic state in morphine microbiome.

Morphine treatment induced simultaneous changes in bacterial and host lipid metabolism are coherent with the increased abundance of various lipid metabolites (ethanolamines, PCs, and FAs) observed in the morphine metabolome. Enrichment of ethanolamines was also observed under various pathological conditions such as in the serum of healthy humans after acute stress, in inflammatory bowel disease (IBD) stool samples, and in colitis model of T-cell transfer.^55,56^ Bacterial species harboring ethanolamine utilization (*Eut B/C*) genes can utilize ethanolamine provided by dead cells as a carbon and nitrogen source, thus providing a growth advantage,^57–60^ and *Eut B/C* genes have been implicated in pathogenesis and colonization of *E. coli* in urinary tract infections and CD.^59,61^ Our metagenomic data indicate an increased abundance of *EutC* gene in *E. faecalis*, thus might provide it with a competitive advantage over commensal microbiota. Also, N-acylethanolamines (NEAs) augmentation led to the expansion of pathogenic *E. coli*, *E. faecalis*, and *Ruminococcus gnavus* and resulted in the shift of complex ex-vivo microbial cultures to a more IBD like microbiome.^55^ Our integration results are in tandem with this, and NAEs had strong positive correlations to *P. excrementihominis*, *B. bacterium 1_1_47*, *E. faecalis, E. caecimuris* and *A. municiphila, and* DEGs involved in inflammatory response thereby implicating NEAs in the expansion of certain pathogenic bacterial taxa and inflammation.

Accumulation of PCs, another major lipid metabolite group was observed in morphine metabolome. PCs, regulate many biological processes and act as precursors for choline, betaine, and acetylcholine, and influence DNA methylation through one-carbon pathway.^62^ Importantly, increased abundance of PCs was observed in people with opioid use disorder compared to opioid users.^14^ Gut bacterial metabolism of choline is implicated in the production of trimethylamine o-oxide (TMAO), a harmful metabolite.^63,64^ Also, bacterial utilization of choline influences the choline availability to host and thus affect the DNA methylation levels and had transgenerational effect on development of anxiety.^65^ Considering the high incidence of opioid use in pregnant population and females of childbearing age, and evidence of microbial dysbiosis in mothers and neonates prenatally exposed to hydromorphone,^4,13^ future studies dissecting dysbiotic microbiome mediated changes in PC metabolism in line with TMAO production, and changes in DNA methylation are much needed.

Furthermore, a decrease in riboflavin was observed in morphine metabolome. Decreased riboflavin levels were also noted in patients using opioids.^66^ Riboflavin has anti-inflammatory property and its supplementation was shown to inhibit the activation of inflammasome NLRP3,^67^ protect from LPS-induced septic shock,^68^ enhance phagocytosis, and limit inflammation.^69^ Consistently, depletion of riboflavin negatively correlated with several inflammatory response genes such as *Tlr2*, *Il6*, *Il18*, *Nlrp12*, *Ccl22*, *Cxcr2* and *Ccl17*. Riboflavin is vital for the maintenance of redox potential, protein synthesis, and survival of microbes,^70^ and thus, as an adaptive mechanism, an increase in the riboflavin biosynthesis and transporter genes was observed in morphine microbiome. However, other bacterial taxa which lack the genomic capability to produce riboflavin, including *L. johnsonii NCC335*,^71^ a morphine mediated decrease in riboflavin availability can be detrimental. Correspondingly, in our integration analysis, depletion of riboflavin positively correlated with depletion of *L. johnsonii* and *Peptostreptococcaceae* and negatively correlated with expansion of *P. excremntihominis, B. bacterium* and *E. caecimuris*. Riboflavin supplementation in humans was shown to modulate microbiome.^72^ Riboflavin in the form of flavin adenine dinucleotide and flavin mononucleotide participates in deactivation of reactive oxygen species (ROS).^70^ Our results and of others indicate that morphine treatment results in inflammatory milieu, activation of macrophages, and influx of neutrophils, which is associated with increased production of ROS and oxidative stress.^73^ An increased expression of genes *Cox1*, *Duoxa1*, *Duoxa2*, *Nos1*, *Nos2*, and *Nos3* was indicative of increased oxidative stress in intestinal tissue of morphine treated mice. The increased oxygen radical could favor the expansion of facultative anaerobic bacteria at the expense of obligate anaerobes as observed in morphine dysbiotic bacteria.

In addition to riboflavin, other metabolite changes indicate a decrease in antioxidant levels in the morphine treatment groups, including depletion of spermidine, phenylpropionate, and flavonoids. Flavonoids are shown to improve barrier function and limit inflammation.^74^ These findings suggest a potential use for riboflavin and flavonoids as potent nutritional targets and need to be tested in preclinical models of opioid use.

Additionally, BAs are shown to regulate the gut mucosal immune responses. We observed downregulation of bile acids transporter genes *Asbt*, *Ostα*, and *Ostβ* which can directly influence the bioavailability of bile acid to intestinal immune cells. Vitamin A metabolite, *at*RA plays a crucial role in modulating adaptive mucosal immunity and gut tropism in lymphocytes. Vitamin A in the form of retinal is metabolized to *at*RA by retinol dehydrogenase (RDH) and retinal dehydrogenases in small intestine. Morphine-induced depletion of *at*RA negatively correlated with genes involved in cellular response to cytokine stimulus, inflammatory response, and TH1 type immune response, signifying the role of *at*RA in maintaining mucosal immune homeostasis. Alongside, we observed a positive correlation between *at*RA and morphine depleted commensal *L. johnsonii*. Although the literature on the role of the gut microbiome on RA metabolism is sparse, recently commensal bacterial taxa from class Clostridia is shown to modulate RA concentration by suppressing the expression of *Rdh7*.^75^ We observed a significant decrease in expression of *Rdh7* gene in morphine treated group compared to the AM group further confirming the role of microbiome in retinoic acid metabolism.

Together, these findings further highlight the roles of dysbiotic microbiome, alterations in bacterial and host lipid metabolism riboflavin, and flavonoids, etc., in regulating inflammation and host physiology. It is imperative to further understand the relationship between opioid induced alterations in lipid metabolism and opioid associated gut pathology. This study also provided an understanding of the bidirectional host microbiome interactions. However, there are a few limitations to the current study. Firstly, some of these associations remain correlative and warrant further experimental confirmation. Another limitation of the study is the use of an untargeted metabolomics approach, which led to a measure of relative abundances and not absolute concentrations of metabolites. Although we haven’t measured systemic changes in metabolites in blood/serum, the luminal metabolite would serve as a proxy for serum measures. Lastly, even though our metagenomic data have identified significant changes in methanogenic archaea *Methanococcus maripaludis*, fungi, and viruses in the morphine microbiome, the data is still in its infancy and the functional consequences of these taxonomical changes were not assessed and further studies are warranted to understand their role in production of metabolites and morphine pathophysiology. Our data implies a novel therapeutic intervention targeting ethanolamine metabolism to limit deleterious changes in the gut microbiome following opioid use. Our study also suggests that NEA and PCs can be used as potential biomarkers for intestinal damage and inflammation. In addition, we have identified riboflavin and flavonoids as potent nutritional targets to limit dysbiosis and inflammation. Data from our study hence provide much-needed groundwork and aids in the identification of biomarkers and targets for therapeutic interventions against opioid associated comorbidities and to help understand human studies where mechanistic work such as this is impossible.

## Abbreviation

LEfSe, linear discriminant analysis of effect size; LDA, linear discriminant analysis; FDR, false discovery rate; GO, gene ontology; KOs, KEGG orthologs; PCoA, Principial Coordinate Analysis; LC-MS, Liquid chromatography-mass spectrometry; PC, phosphocholines; FA, Fatty acids; M6G, morphine 6-glucoronide; NEA, N-acylethanolamines; DEGs, differentially expressed genes; FC, fold change; GF, germ free; LPS, lipopolysaccharides; LTA, lipoteichoic acid; CD, Crohn’s disease; IBD, inflammatory bowel disease; Eut, ethanolamine utilization; TMA, trimethylamine; TMAO, Trimethylamine O-oxide; ROS, reactive oxygen species; BSH, bacterial bile salt hydrolase; RDH, retinol dehydrogenase; *at*RA, all-trans-retinoic acid.

## Supplementary Material

Supplemental Material

## Data and material availability

The data that support the findings of the study are available in Genbank repository at https://www.ncbi.nlm.nih.gov/geo/query/acc.cgi?acc=GSE230096 with reference number “GSE230096”.
