# Supplementary material for: Multi-omics analysis revealing the interplay between gut microbiome and the host following opioid use
Source: Gut Microbes. 2023 Aug 23;15(2):2246184. doi: 10.1080/19490976.2023.2246184 (PMC10448978; doi:10.1080/19490976.2023.2246184)
Supplement: Supplemental Material [file KGMI_A_2246184_SM4472.zip › Supplemental material_KGMI 2246184/Supplementary Figure Legends.docx]

**Supplementary Figure 1: Study design and overview.** Mice (8-12 weeks) were treated with and without morphine for 16 hrs. Ileal tissue sample was utilized for RNA-sequencing, Ileal luminal content for metabolite analysis and metagenomic shot gun sequencing. To understand the role of microbiome in mediating morphine pathology, a group of animals were treated with and without pan antibiotics cocktail to deplete microbiome and then treated with morphine. Ileal tissue was utilized for gene expression changes using RNA-seq.

**Supplementary Figure 2:** **Interkingdom level microbial dysbiosis in context of morphine treatment.** (**A**) Morphine treatment increased the bacterial richness as indicated by Shannon (p=0.016) and Simpson (p=0.009) alpha diversity box plots. (**B**) PCoA plot using Bray Curtis distance on bacterial taxa showing separate clustering after morphine treatment. (**C**) Box plot showing significant changes in relative abundance of selected archaea and bacterial species between control and morphine groups. (**D**) Morphine treatment cause increased expression of virulence factor genes in in *Bacteroides fragilis* as shown by box plots. Morphine treated mice did not show any significant change in virus community of gut microbiome as shown by (**E**) Bray-Curtis PCoA plot and (**F**) Simpson and Shannon index. n=5 per group. Data (C, D) were analyzed using Mann – Whitney U test; the box-and-whisker plot indicates the minimum and maximum distribution (whiskers) and the upper and lower quartile limits (box), with the median value shown as a line. *p ≤ 0.05, **p ≤ 0.01, ***p ≤ 0.001, ****p ≤ 0.0001.

**Supplementary Figure 3: Functional changes in metagenomic data** **in morphine and control microbiome.** Heat map showing 201 KEGG orthologs (KOs) significantly altered after morphine treatment. Data were analyzed by mapping microbial reads using HUMAnN pipeline and assigning identified genes to KOs.

**Supplementary Figure 4:** **Gastrointestinal changes in mice model of opioid use.** (**A**) Representative H&E-stained intestinal section from morphine group showed severe damage to intestinal morphology compared with control group. (**B**) Representative image showing Claudin-1 tight junction staining in small intestinal section of morphine and control mice. (**C**) Bar graph showing changes in inflammatory markers between control and morphine group using qPCR. n=5 per group. Data were analyzed using Mann – Whitney U test. (Mean± standard error of mean (SEM)) *p ≤ 0.05, **p ≤ 0.01, ***p ≤ 0.001, ****p ≤ 0.0001.

**Supplementary Figure 5: Morphine treatment in** **germ-free mice confirms the role of gut microbiota in driving immune related and tissue damage related gene changes.** No significant change was observed in selective cytokines (IL6, IL1β, IL18, TNF α), chemokines (CXCL5, CXCL17) and matrix metallopeptidases (MMP13, MMP16) involved in tissue damage in morphine treated germ free mice compared to control germ free mice. n=5 per group. Data were analyzed using Mann – Whitney U test. (Mean± SEM)

**Supplementary Figure 6:** **Multi-omic integration of microbiome, metabolome, and host transcriptome**. Sub-network showing significant correlations between metabolite N-acylethanolamines (NAE) and (A) significantly changed bacterial species (B) differentially expressed inflammatory genes in morphine group. Microbial species, metabolites, and DEGs are represented as nodes; microbiome (square), metabolite (oval), DEGs (triangle). Lines represent statistically significant correlations and are colored red for positive and blue for negative correlations
