# Supplementary figures and images for: Multi-omics analysis revealing the interplay between gut microbiome and the host following opioid use
Source: Gut Microbes. 2023 Aug 23;15(2):2246184. doi: 10.1080/19490976.2023.2246184 (PMC10448978; doi:10.1080/19490976.2023.2246184)

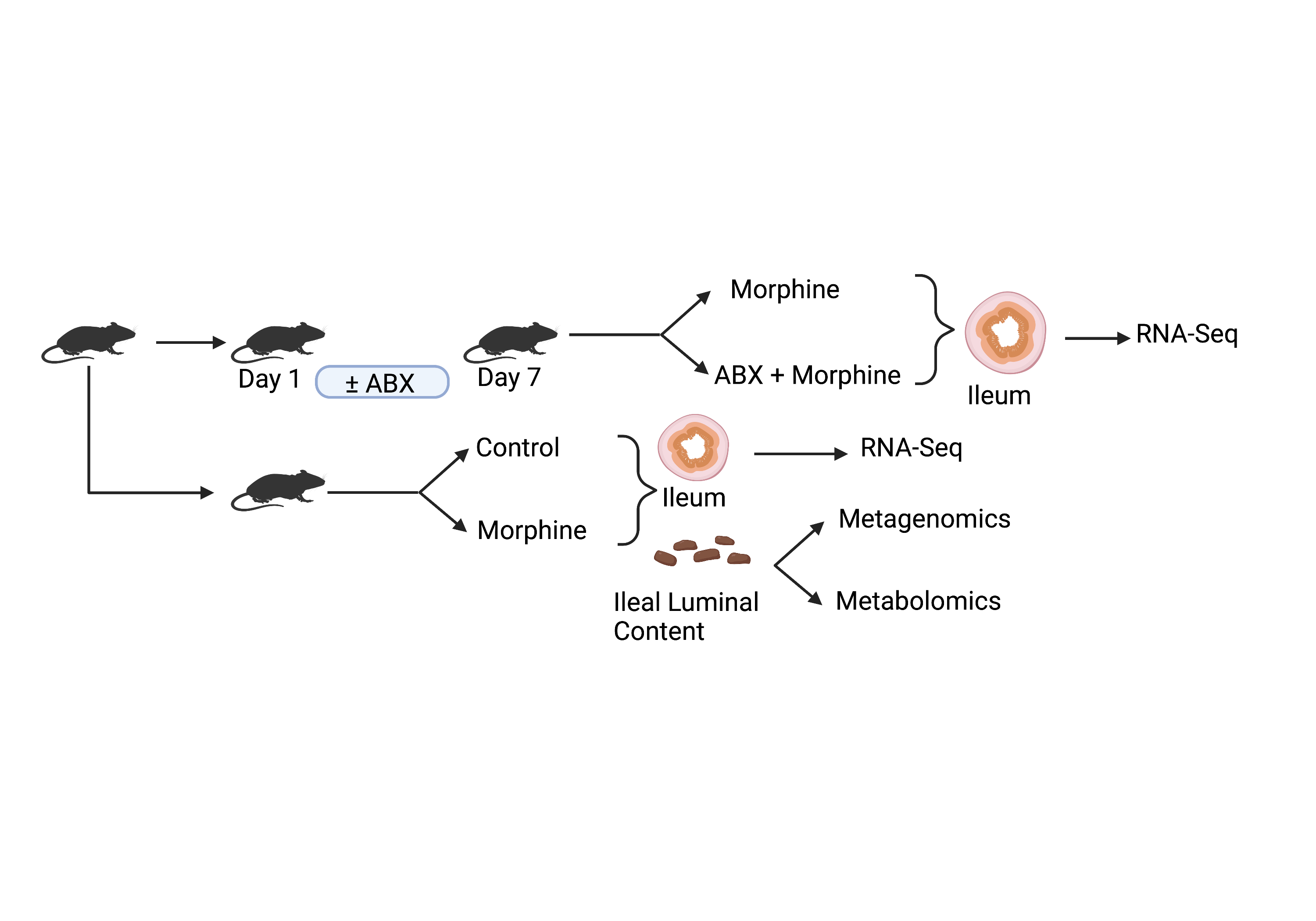

Supplement: Supplemental Material [file KGMI_A_2246184_SM4472.zip › Supplemental material_KGMI 2246184/Supplementary figure 1_revision.tif]

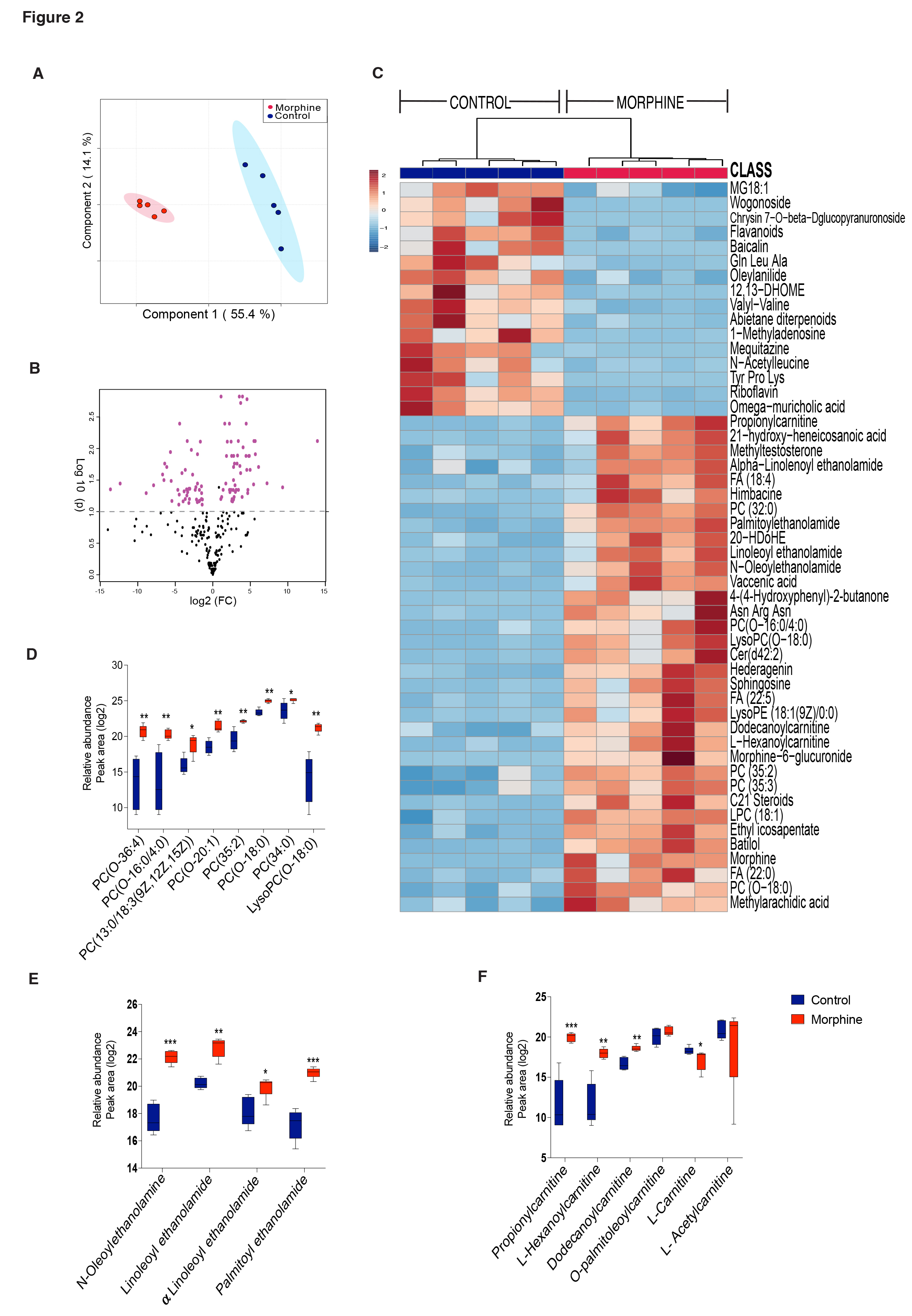

Supplement: Supplemental Material [file KGMI_A_2246184_SM4472.zip › Supplemental material_KGMI 2246184/Supplementary Figure 2 _revision.tif]

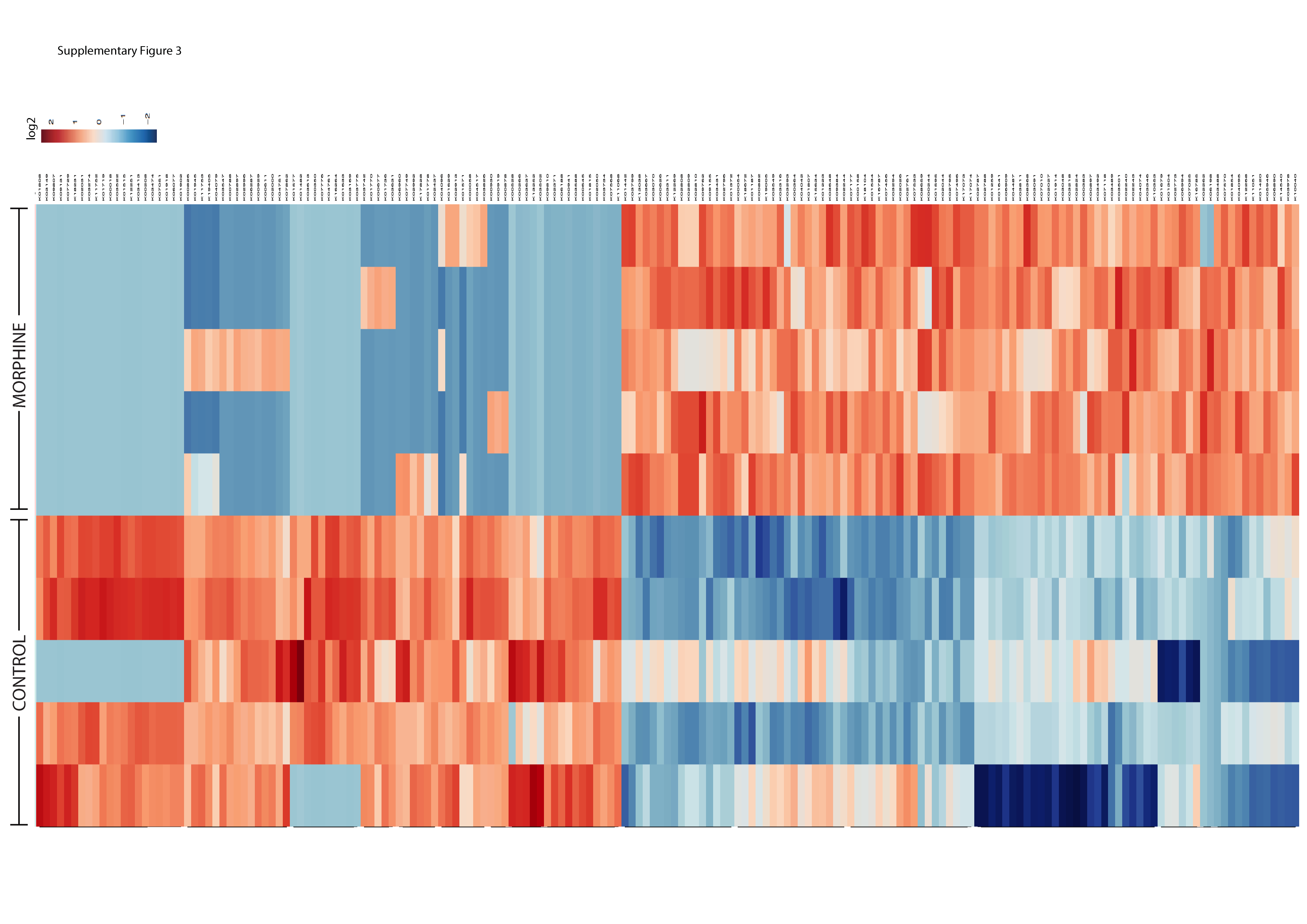

Supplement: Supplemental Material [file KGMI_A_2246184_SM4472.zip › Supplemental material_KGMI 2246184/Supplementary Figure 3_revision .tif]

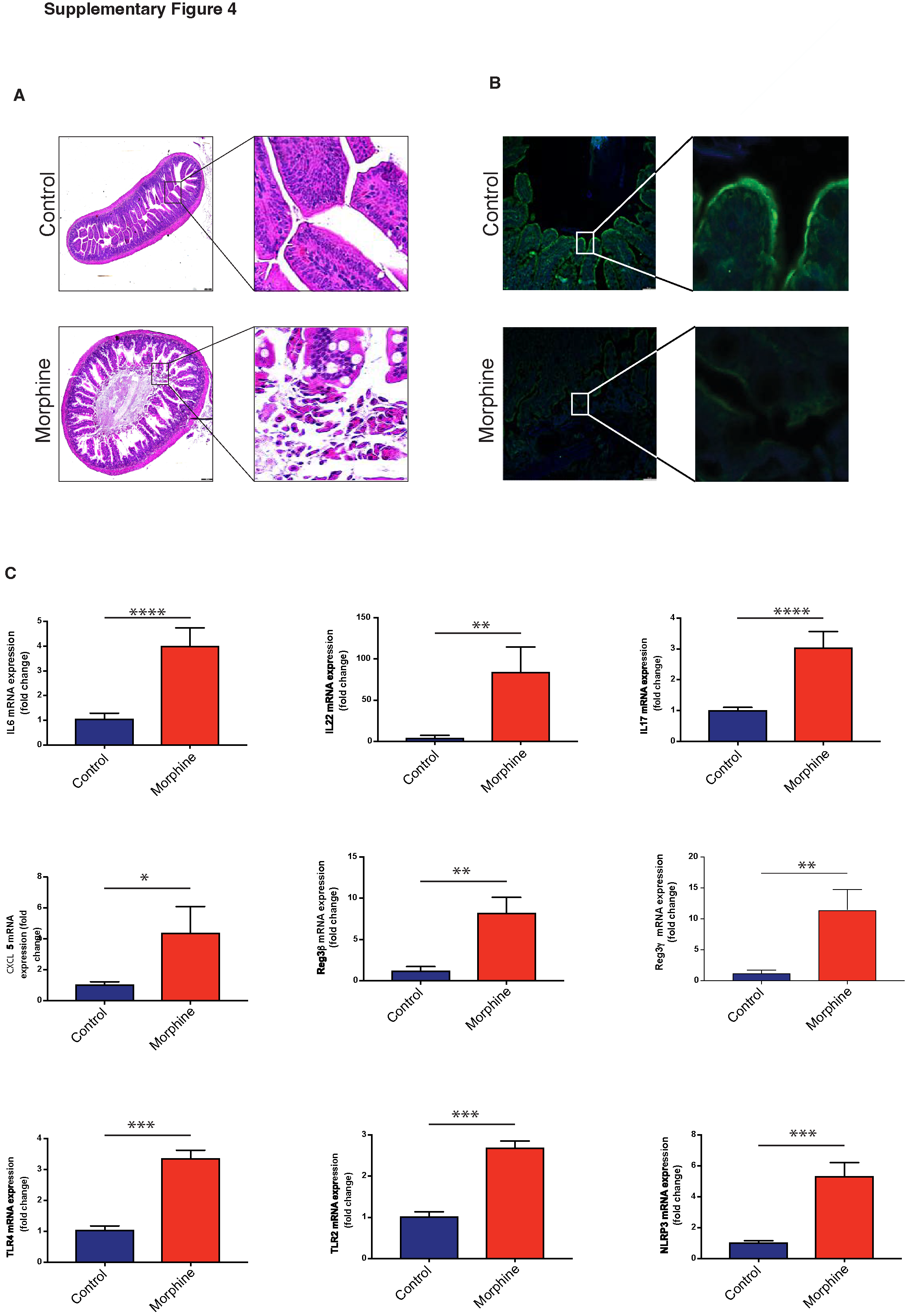

Supplement: Supplemental Material [file KGMI_A_2246184_SM4472.zip › Supplemental material_KGMI 2246184/Supplementary Figure 4_revision.tif]

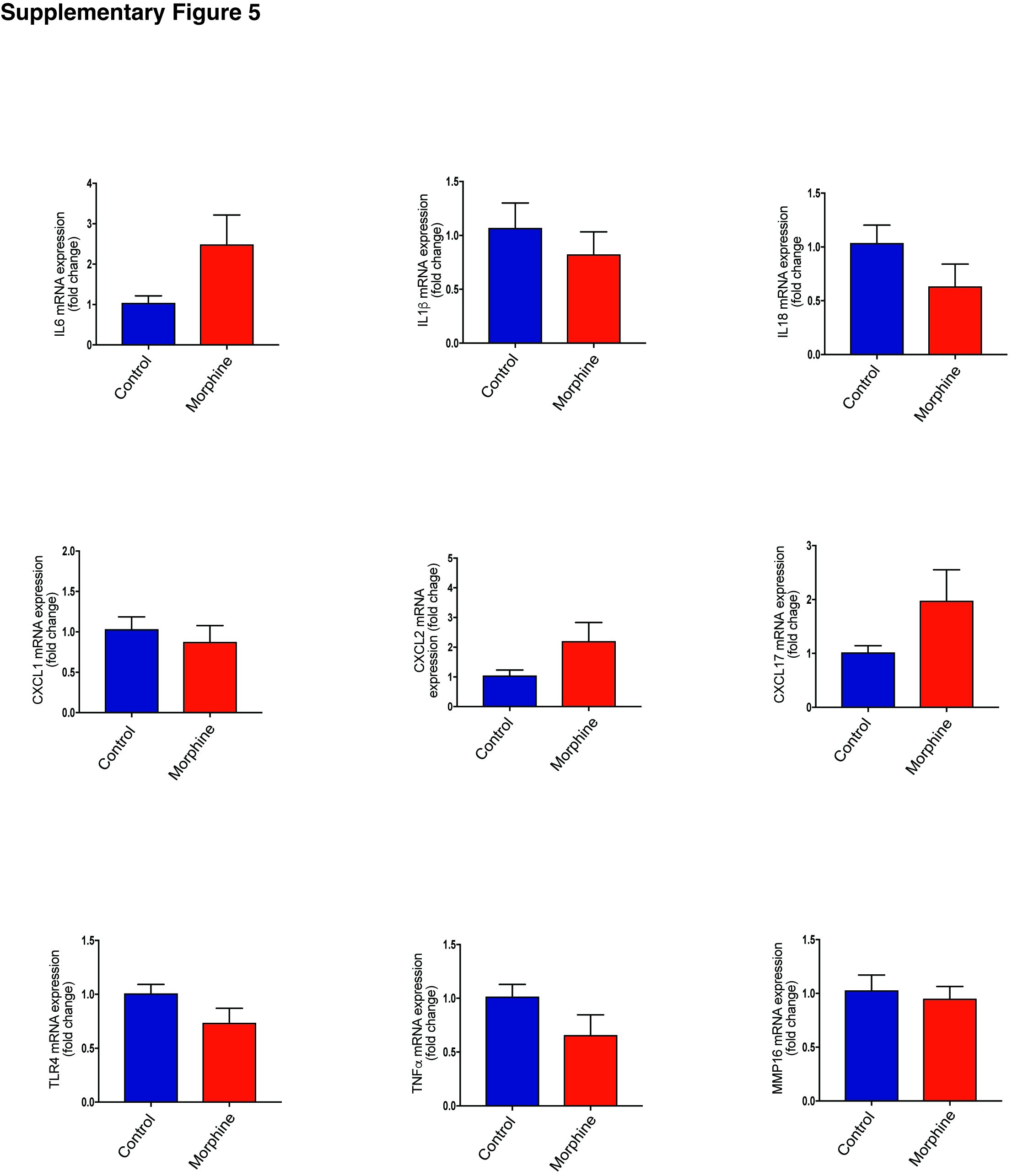

Supplement: Supplemental Material [file KGMI_A_2246184_SM4472.zip › Supplemental material_KGMI 2246184/Supplementary Figure 5_revision.tif]

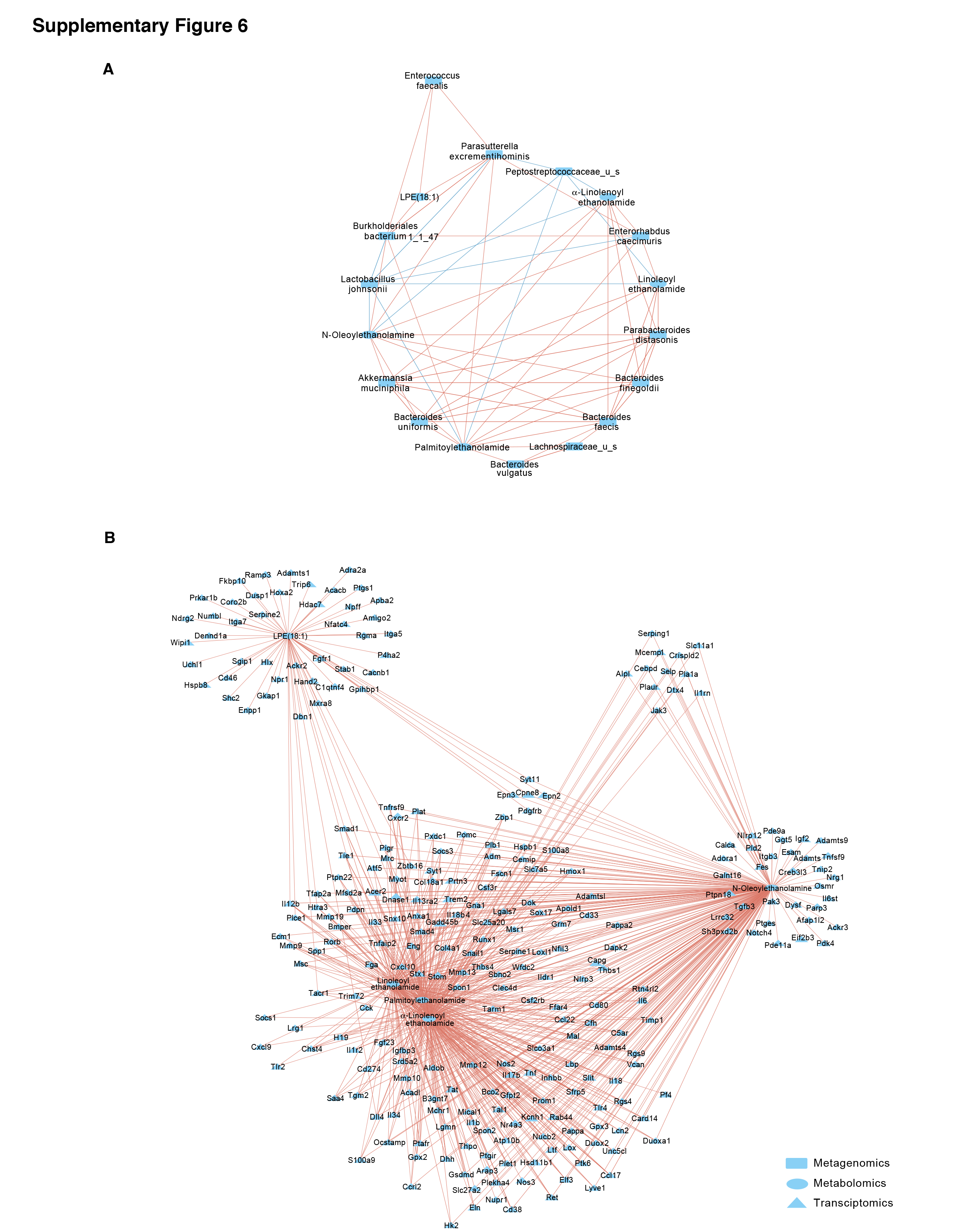

Supplement: Supplemental Material [file KGMI_A_2246184_SM4472.zip › Supplemental material_KGMI 2246184/Supplementary Figure 6_revision.tif]
